# Bcl-2 Proteins Regulate Mitophagy in Lipopolysaccharide-Induced Acute Lung Injury via PINK1/Parkin Signaling Pathway

**DOI:** 10.1155/2020/6579696

**Published:** 2020-02-20

**Authors:** Zhihao Zhang, Zhugui Chen, Ruimeng Liu, Qingchun Liang, Zhiyong Peng, Shuang Yin, Jing Tang, Ting Gong, Youtan Liu

**Affiliations:** ^1^Department of Anesthesiology, Shenzhen Hospital, Southern Medical University, No. 1333, Xinhu Road, Baoan District, Shenzhen, Guangdong Province, 518110, China; ^2^Department of Anesthesiology, Affiliated Hospital of Guangdong Medical University, Renmindadao Road, Xiashan District, Zhanjiang, Guangdong Province, 524002, China; ^3^Department of Anesthesiology, The Third Affiliated Hospital of Southern Medical University, Guangzhou 510000, China

## Abstract

Mitophagy is involved in sepsis-induced acute lung injury (ALI). Bcl-2 family proteins play an important role in mitochondrial homeostasis. However, whether targeting Bcl-2 proteins (Bcl-2 and Bad) could influence mitophagy in ALI remains unclear. In this study, lipopolysaccharide (LPS) was used to induce injury in A549 cells and ALI in mice. LPS treatment resulted in elevated cell apoptosis, enhanced mitophagy, decreased Bcl-2 expression, increased Bad expression, and activation of PINK1/Parkin signaling in cells and lung tissues. Both Bcl-2 overexpression and Bad knockdown attenuated LPS-induced injury, inhibited cell apoptosis and mitophagy, and improved survival. Atg5 knockout (KO) inhibited LPS-induced cell apoptosis. Furthermore, Bcl-2 proteins regulated mitophagy by modulating the recruitment of Parkin from the cytoplasm to mitochondria via direct protein-protein interactions. These results were further confirmed in Park2 KO cells and Park2^−/−^ mice. This is the first study to demonstrate that Bcl-2 proteins regulated mitophagy in LPS-induced ALI via modulating the PINK1/Parkin signaling pathway, promoting new insights into the mechanisms and investigation of therapeutic strategies for a septic patient with ALI.

## 1. Introduction

Acute lung injury (ALI) and acute respiratory distress syndrome (ARDS) contribute to mortality and morbidity in critically ill patients. ALI is a type of diffuse alveolar-capillary membrane injury characterized by pulmonary edema and atelectasis, and ARDS is a serious and life-threatening condition [1, 2]. Studies showed that inflammation, mitochondrial damage, and apoptosis are involved in the etiology of ALI/ARDS [3]. Despite recent progress, the mortality rate associated with ALI/ARDS patients remains high [4].

Mitophagy plays an important role in cell homeostasis by the degradation and removal of damaged mitochondria [5, 6]. However, excessive mitophagy may promote mitochondrial dysfunction and cause cell injury and death [7]. The phosphatase and tension homolog deleted on chromosome 10-induced kinase 1 (PINK1)/Parkin signaling pathway plays a key role in the process of mitophagy. PINK1 shuttles between the cytosol and mitochondria to interact with Parkin, a ubiquitin (Ub) E3 ligase encoded by the Park2 gene [8]. Under normal conditions, PINK1 is degraded by presenilin-associated rhomboid-like protease (PARL). During mitophagy with mitochondrial membrane potential dissipation, the activity of PARL is inhibited, promoting Parkin phosphorylation in the cytoplasm and recruitment to the mitochondria [9]. Polyubiquitinated proteins in the mitochondrial outer membrane trigger the migration of Ub and the LC3 binding adaptor protein p62. This molecule functions to link the damaged mitochondria and mitolysosomes [10, 11], which are formed by the fusion of mitophagosomes and lysosomes to mediate the degradation of the damaged mitochondria [12].

Bcl-2 has been well studied to exert antiapoptotic effects in various apoptotic cell and animal models [13, 14]. Bcl-2 may also regulate autophagy. Hollville et al. showed that Bcl-2 family proteins suppressed mitophagy by inhibiting Parkin translocation to depolarized mitochondria in Hela and HEK293T cells [15]. Bad, an upstream member of the Bcl-2 family, functions as a sensor of cell stress, damage, infection, growth factor deprivation, and apoptotic process [16]. Bad is a BH3-like protein that interacts with Bcl-2. The BH1-BH4 domain of Bcl-2 forms a hydrophobic groove structure and binds to Bad [17].

In this study, we evaluated the role of Bcl-2 proteins in the regulation of mitophagy in lipopolysaccharide- (LPS-) treated A549 cells and LPS-induced ALI in mice. We also investigated cell apoptosis and its relationship with mitophagy in LPS-induced ALI. The effects of Bcl-2 overexpression and Bad knockdown on mitophagy and apoptosis were assessed *in vitro* and *in vivo*. Furthermore, Park2 knockout (KO) cells, Agt5 KO cells, and Park2^−/−^ mice were used to investigate the underlying mechanisms.

## 2. Results

### 2.1. LPS-Induced Injury in A549 Cells and ALI in Mice

**Figure 1:**
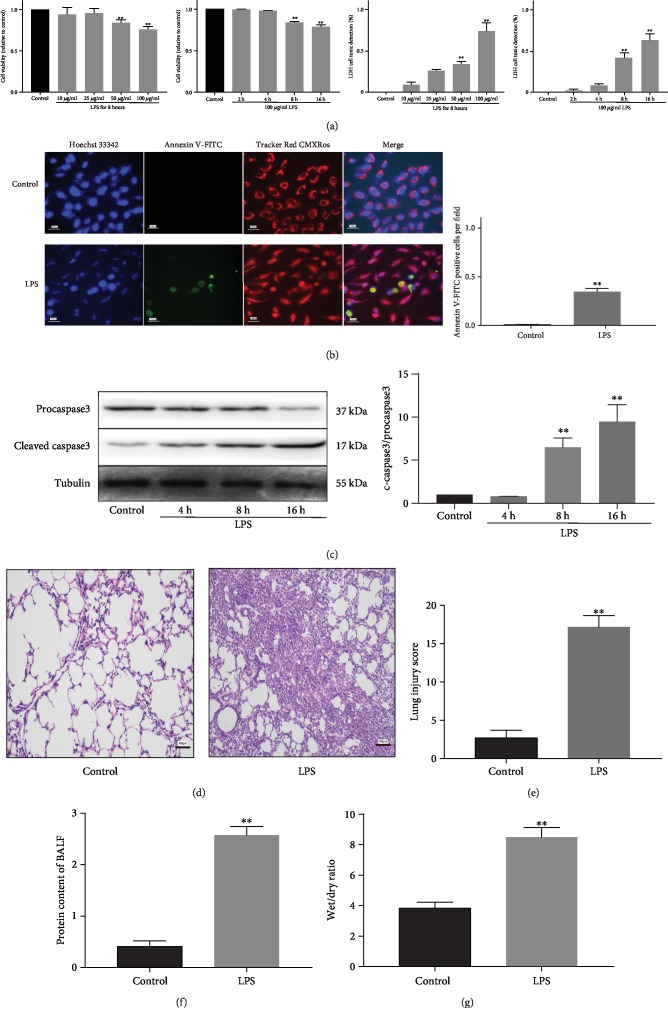
LPS-induced injury in A549 cells and mouse lung tissues. (a) Cell viability and LDH release in A549 cells after exposure to 10–100 *μ*g/ml LPS for 2–16 h (*n* = 6). (b) Hoechst 33342, Annexin V-FITC, and Tracker Red CMXRos staining showing increased cell apoptosis 16 h after exposure to 50 *μ*g/ml LPS (*n* = 3). Data represent mean number of Annexin V-FITC-positive cells in 10 microscopic fields per group. (c) Western blots showing cleaved caspase3 expression in A549 cells after 4, 8, and 16 h of LPS exposure (*n* = 3). (d) HE staining of mouse lung tissues 24 h after intratracheal injection of 5 mg/kg LPS.(e) Increased lung injury score in LPS-treated mice (*n* = 6). (f) Increased protein content in BALF of LPS-treated mice (*n* = 6). (g) Increased wet/dry weight ratio of lungs in LPS-treated mice (*n* = 6). ^∗∗^*P* < 0.01 vs. the control group. BALF: bronchoalveolar lavage fluid.

CCK-8 and LDH assays showed significantly decreased cell viability and increased cell toxicity in A549 cells treated with 50 and 100 *μ*g/ml LPS for 8 and 16 h (Figure 1(a)). We selected 50 *μ*g/ml LPS exposure of 16 h for the following experiments. Next, we found LPS treatment led to elevated cell apoptosis (Figure 1(b)). The ratio of cleaved caspase3 to procaspase3 protein expression was significantly increased 8 and 16 h after LPS exposure (Figure 1(c)).

In mice, LPS induced inflammatory cell infiltration in the alveolar and connective tissue of the alveolar septum, with significant neutrophil aggregation around the pulmonary vessels and bronchi (Figure 1(d)). Lung tissues showed hyperemia, edema, alveolar diaphragm thickening, and early transparent membrane formation in the alveolar wall surface. In addition, increased inflammatory cells were found in the alveolar lavage fluid in LPS mice (Figure 1(d)). Lung injury scores were then calculated using the pathological ALI scoring system according to the American Thoracic Association criteria [18]. Compared with the control group, the LPS group showed significantly a higher lung injury score (Figure 1(e)), BALF protein content (Figure 1(f)), and wet/dry ratio (Figure 1(g)).

### 2.2. LPS-Induced Mitophagy in A549 Cells and Mouse Lung Tissues

**Figure 2:**
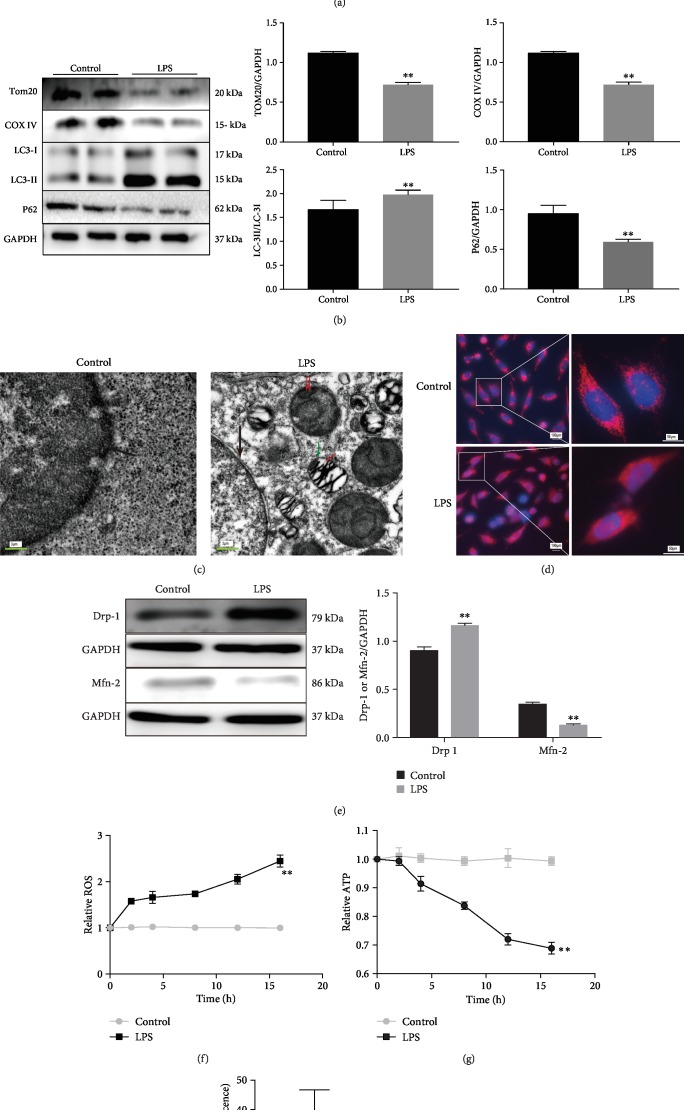
LPS-induced mitophagy in A549 cells and mouse lung tissues. (a) Immunofluorescence imaging of control and LPS-treated A549 cells transfected with GFP-LC3 and mito-dsred adenovirus. (b) Western blots showing expression of Tom20, COX IV, LC3-I, LC3-II, and P62 proteins (*n* = 3). (c) Transmission electron microscopy showing increased mitophagy in LPS-treated mice. Black arrow, nucleus; green arrow, mitochondria; red arrow, mitophagosome; and double red arrow, mitolysosome. (d) Hoechst 33342 and Tracker Red CMXRos staining showing damaged mitochondrial reticular structure in LPS-treated cells. (e) Western blots showing increased Drp1 and decreased Mfn2 protein expression in LPS-treated cells (*n* = 3). (f, g) Increased mito-ROS and decreased ATP over time in LPS-treated cells (*n* = 3). (h) JC-1 assay showing dissipated mitochondrial membrane potential in LPS-treated cells (*n* = 6). ^∗∗^*P* < 0.01 vs. the control group.

In LPS-treated cells, we observed increased colocalization of LC3GFP and MitoTracker red (MTR) (Figure 2(a)). LPS resulted in deceased expression of mitochondrial outer membrane protein Tom20, mitochondrial inner membrane protein COX IV, and P62, with an increased LC3-II/LC3-I ratio (Figure 2(b)). Electron microscopic images showed increased mitophagy induced by LPS [19] (Figure 2(c)). We also found that LPS caused remarkable damage to the mitochondrial network and structure (Figure 2(d)). Under stress conditions, damaged mitochondria are isolated from the rest of the mitochondrial network or repaired via mitochondria fusion [20], which was regulated by a number of mitophagy-related proteins [21]. Mitofusin-2 (Mfn2) plays a critical role in mitochondrial fusion [22], while dynamin-related protein 1 (Drp1) promotes mitochondrial fission [21]. In this study, the protein expression of Mfn2 was reduced and Drp1 expression was increased by LPS (Figure 2(e)). In addition, LPS exposure significantly increased reactive oxygen species (ROS) levels (Figure 2(f)) and decreased ATP levels (Figure 2(g)) and resulted in decreased mitochondrial membrane potential (Figure 2(h)). These findings suggest that the activation of mitophagy plays a critical role in LPS-induced ALI.

Moreover, we used CRISPR-Cas9 gene editing technology to knock out the Atg5 gene, an important factor of mitophagy, in A549 cells ([Supplementary-material supplementary-material-1]). Knockout of Atg5 has been indicated to inhibit mitophagy [23]. Our results showed that apoptosis induced by LPS was alleviated in Atg5 KO cells compared to wild-type cells (Figures [Supplementary-material supplementary-material-1] and [Supplementary-material supplementary-material-1]). Therefore, inhibition of mitophagy could attenuate apoptosis in LPS-induced ALI.

### 2.3. Bcl-2 Family Proteins and PINK1/Parkin Signaling during LPS Exposure

**Figure 3:**
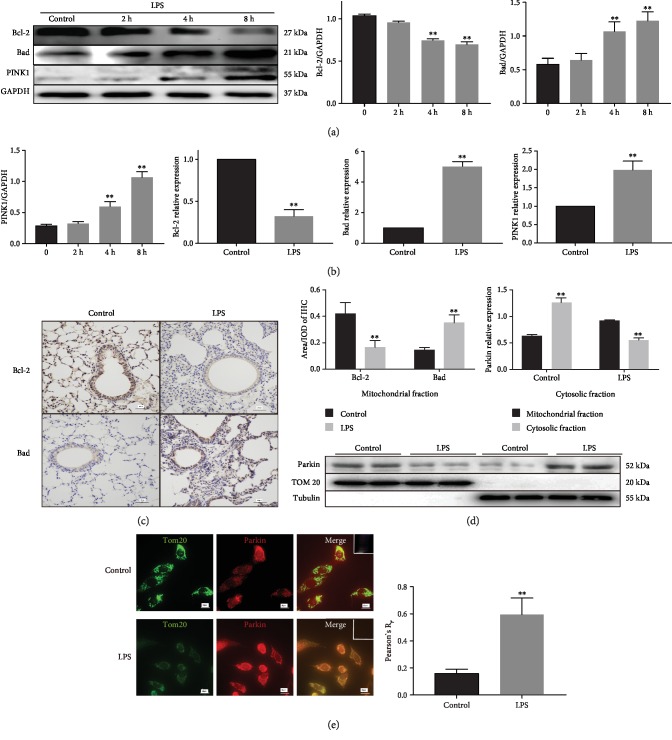
LPS-induced changes in the expression of Bcl-2, Bad, and PINK1/Parkin signaling *in vitro* and *in vivo*. (a) Western blots showing the expression of Bcl-2, Bad, and PINK1 in A549 cells at 2, 4, and 8 h after LPS exposure (*n* = 3). (b) RT-qPCR showing the mRNA expression of Bcl-2, Bad, and PINK1 in cells at 8 h after LPS exposure (*n* = 3). (c) Immunohistochemistry showing the expression of Bcl-2 and Bad in lung tissues of control and LPS-treated mice (*n* = 6). (d) Western blots showing Parkin protein expression in the mitochondria and cytoplasm of control and LPS-treated cells (*n* = 3). (e) Immunofluorescence showing the colocalization of Tom20 (green) and Parkin (red) and Pearson correlation analysis showing high association between Tom20 and Parkin in the LPS group (*n* = 3). ^∗∗^*P* < 0.01 vs. the control group.

Western blot and RT-qPCR analyses showed that LPS exposure led to decreased Bcl-2 expression and increased expression of Bad and PINK1 at 4 and 8 h in A549 cells (Figures 3(a) and 3(b)). We also found downregulated Bcl-2 expression and upregulated Bad expression in LPS-induced ALI tissues (Figure 3(c)). By isolating the mitochondrial and cytoplasmic components of A549 cells, LPS-treated cells showed decreased Parkin expression in the cytoplasm and increased Parkin expression in the mitochondria (Figure 3(d)). In addition, we found increased expression and colocalization of Parkin and Tom20 during LPS treatment (Figure 3(e)). The colocalization of Parkin and Tom20 was evaluated by the calculation of Pearson's correlation coefficient [24]. In line with our results, a previous study showed that Parkin is recruited from the cytoplasm to the dysfunctional mitochondria during mitochondrial depolarization [25]. Besides, the protein expression of other members of Bcl-2 family proteins including Bcl-xl, Bcl-w, and Bim was assessed. LPS treatment significantly decreased Bcl-xl and Bcl-w expression and increased Bim expression ([Supplementary-material supplementary-material-1]). These findings suggest the involvement of Bcl-2 family proteins and enhanced PINK1/Parkin-mediated mitophagy during LPS treatment.

### 2.4. Bcl-2 Overexpression Attenuated Cell Injury In Vitro and ALI In Vivo by Inhibiting Mitophagy

**Figure 4:**
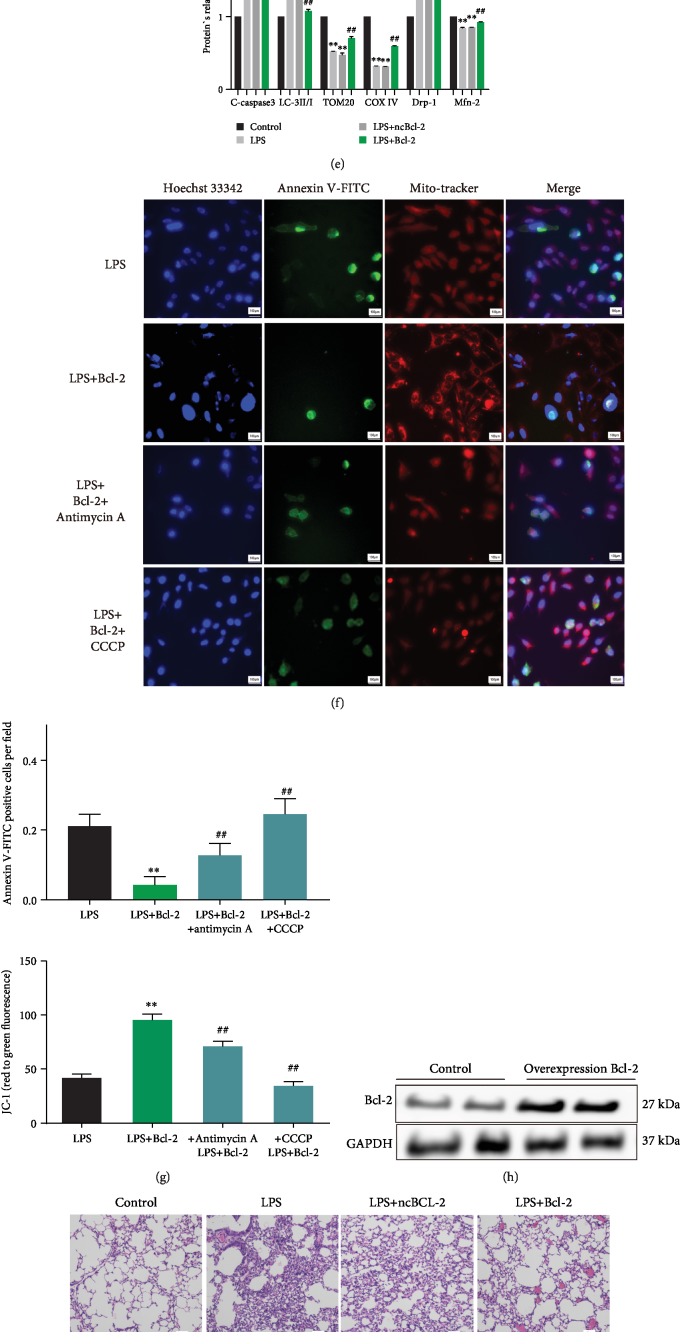
Bcl-2 overexpression in LPS-treated A549 cells and mice. (a) Western blots showing Bcl-2 protein expression in A549 cells after transfection with Bcl-2 overexpression adenovirus for 48 h. (b) CCK-8 assay showing that Bcl-2 overexpression increased cell viability in LPS-treated cells (*n* = 3). (c, d) Bcl-2 overexpression increased total ATP and reduced mito-ROS in LPS-treated cells (*n* = 3). (e) Western blots showing the expression of cleaved caspase3, LC3-I, LC3-II, Tom20, COX IV, Drp1, and Mfn2 in the control, LPS, LPS+ncBcl-2, and LPS+Bcl-2 groups (*n* = 3). (f) Annexin V-FITC staining showing cell apoptosis after administration of the mitochondrial autophagy inducer antimycin A or CCCP (*n* = 3). (g) JC-1 assay showing mitochondrial membrane potential in LPS-treated cells following administration of antimycin A or CCCP (*n* = 3). (h) Western blots showing Bcl-2 protein expression in mice injected with the Bcl-2 overexpression adenovirus. (i, j) HE staining showing that Bcl-2 overexpression reduced LPS-induced lung injury in mice (*n* = 6). (k) Transmission electron microscopy showing mitophagy in mouse lung tissues. Black arrow, nucleus; green arrow, mitochondria; red arrow, mitophagosome; and double red arrow, mitolysosome. (l) Bcl-2 overexpression reduced the wet/dry weight ratio in LPS-treated mice (*n* = 6). ^∗∗^*P* < 0.01 vs. the LPS group (b–d, f, g) or vs. the control group (e, j, l); ^##^*P* < 0.01 vs. the LPS group (e, j, l) or vs. the LPS+Bcl-2 group (f, g).

To explore the role of Bcl-2 in LPS-induced ALI, overexpression of Bcl-2 was carried out by transient transfection of A549 cells with the AAV-CMV-Bcl-2 vector (Figure 4(a)). Bcl-2 overexpression significantly increased cell viability (Figure 4(b)), restored total ATP (Figure 4(c)), and reduced ROS levels (Figure 4(d)) in LPS-treated cells. Reduced expression of cleaved caspase3 (Figure 4(e)) and Annexin V-FITC staining (Figure 4(f)) indicated that LPS-induced apoptosis was significantly alleviated by Bcl-2 overexpression. Bcl-2 overexpression also restored the expression of Tom20, COX IV, and mitofusin-2 (Mfn2) and inhibited dynamin-related protein 1 (Drp1) expression and LC3-II/LC3-I ratio (Figure 4(e)). Moreover, the reduced apoptosis was reversed by the use of mitophagy inducer antimycin A (500 nM) or CCCP (5 *μ*M) (both from Selleck Chemicals, Houston, TX, USA) (Figure 4(f)). These findings suggest that Bcl-2 attenuates LPS-induced injury by inhibiting mitophagy. Bcl-2 family members have been shown to be associated with the remodeled mitochondrial networks [21]. JC-1 assays showed that Bcl-2 overexpression alleviated LPS-induced mitochondrial membrane potential dissipation, which was blocked by antimycin A or CCCP (Figure 4(g)).

The effects of Bcl-2 overexpression were also investigated in mice (Figure 4(h)). HE staining showed that Bcl-2 overexpression significantly reduced the inflammatory cell infiltration in lung tissues (Figure 4(i)) and improved lung injury scores (Figure 4(j)) during LPS-induced ALI. Furthermore, transmission electron microscopy showed fewer mitophagosomes in the Bcl-2 overexpression group compared to the control group (Figure 4(k)). Bcl-2 overexpression also reduced the wet/dry weight ratio in LPS-induced ALI tissues (Figure 4(l)). These findings suggest that LPS resulted in mitochondrial dysfunction, apoptosis, and excessive mitophagy, which was attenuated by Bcl-2 overexpression.

### 2.5. Bad Knockdown Attenuated Cell Injury In Vitro and ALI In Vivo by Inhibiting Mitophagy

**Figure 5:**
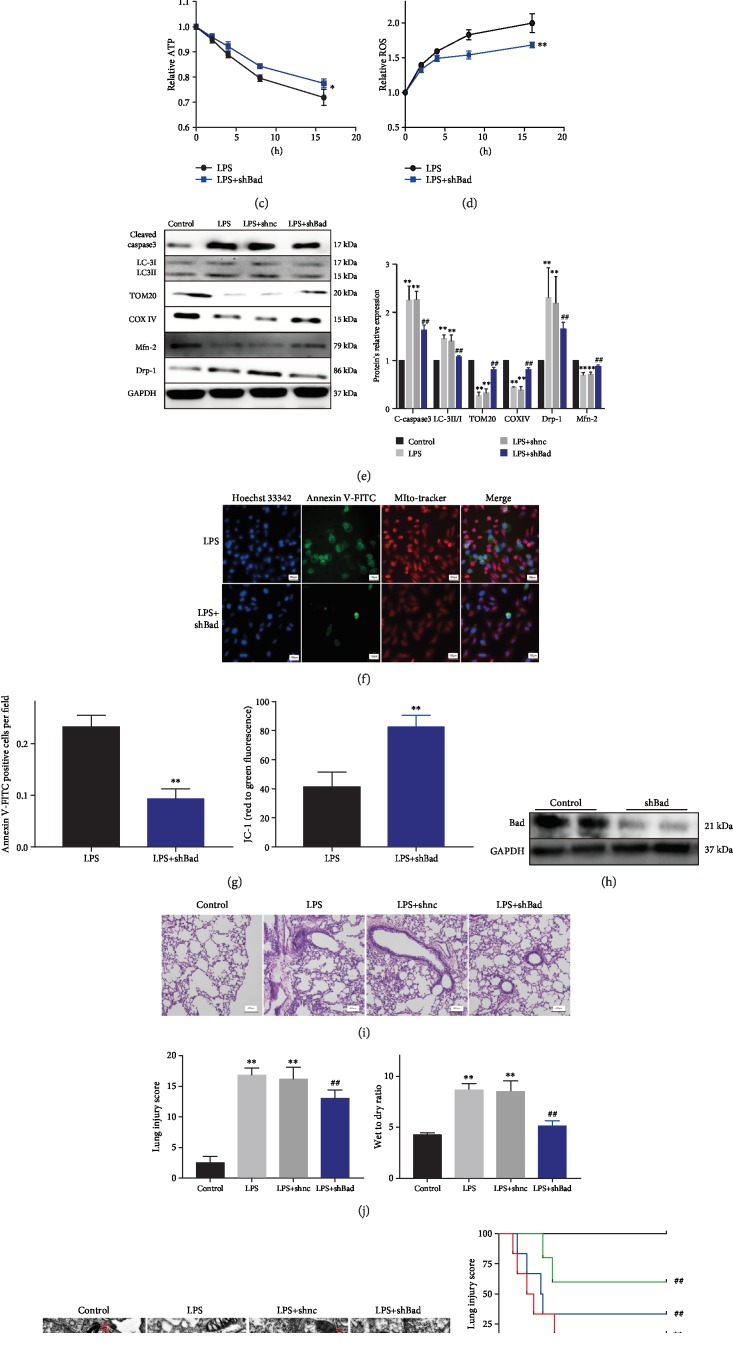
Bad knockdown in LPS-treated A549 cells and mice. (a) Western blots showing Bad protein expression in A549 cells after transfection with shBad lentivirus for 48 h. (b) CCK-8 assay showing that shBad increased viability of LPS-treated cells (*n* = 3). (c, d) Bad knockdown with shBad increased total ATP and reduced mito-ROS in LPS-treated cells (*n* = 3). (e) Western blots showing the expression of cleaved caspase3, LC3-I, LC3-II, Tom20, COX IV, Drp1, and Mfn2 in the control, LPS, LPS+shnc, and LPS+shBad groups (*n* = 3). (f) Annexin V-FITC staining showing inhibition of cell apoptosis by shBad (*n* = 3). (g) JC-1 assay showing that shBad increased mitochondrial membrane potential (*n* = 3). (h) Western blots showing Bad protein in mice injected with shBad lentivirus. (i) HE staining showing that shBad reduced LPS-induced lung injury in mice (*n* = 6). (j) shBad reduced the wet/dry weight ratio in LPS-treated mice (*n* = 6). (k) Transmission electron microscopy showing mitophagy in mouse lung tissues. Black arrow, nucleus; green arrow, mitochondria; red arrow, mitophagosome; and double red arrow, mitolysosome. (l) Survival analysis up to 72 h of mice in the control, LPS, LPS+Bcl-2 overexpression, and LPS+shBad groups (*n* = 6). ^∗^*P* < 0.05 and ^∗∗^*P* < 0.01 vs. the LPS group (b–d, f, g) or vs. the control group (e, i, j); ^##^*P* < 0.01 vs. the LPS group.

To evaluate the role of Bad in LPS-induced ALI, Bad knockdown was achieved by transfection with shBad lentivirus (Figure 5(a)). Bad knockdown increased cell viability (Figure 5(b)) and total ATP (Figure 5(c)) and reduced ROS levels (Figure 5(d)). Bad knockdown also reduced the LC3-II/LC3-I ratio and the expression of cleaved caspase3 and Drp1 and restored the expression of Tom20, COX IV, and Mfn2 during LPS-induced cell injury (Figure 5(e)). In addition, Bad knockdown reduced the apoptosis rate (Figure 5(f)) and restored mitochondrial membrane potential during LPS treatment (Figure 5(g)).

In LPS-treated mice, Bad knockdown reduced the lung injury score (Figure 5(i)) and the wet/dry weight ratio (Figure 5(j)). Transmission electron microscopy revealed fewer mitophagosomes in the shBad group compared with the control group (Figure 5(k)). In the survival analysis, both Bcl-2 overexpression and Bad knockdown improved the survival of LPS-treated mice (Figure 5(l)). These findings suggest that Bad protein modulates mitophagy and that Bad knockdown could produce similar protection against LPS-induced mitochondrial dysfunction as Bcl-2 overexpression.

### 2.6. Bcl-2 Proteins Regulate Parkin Recruitment from the Cytoplasm to Mitochondria

**Figure 6:**
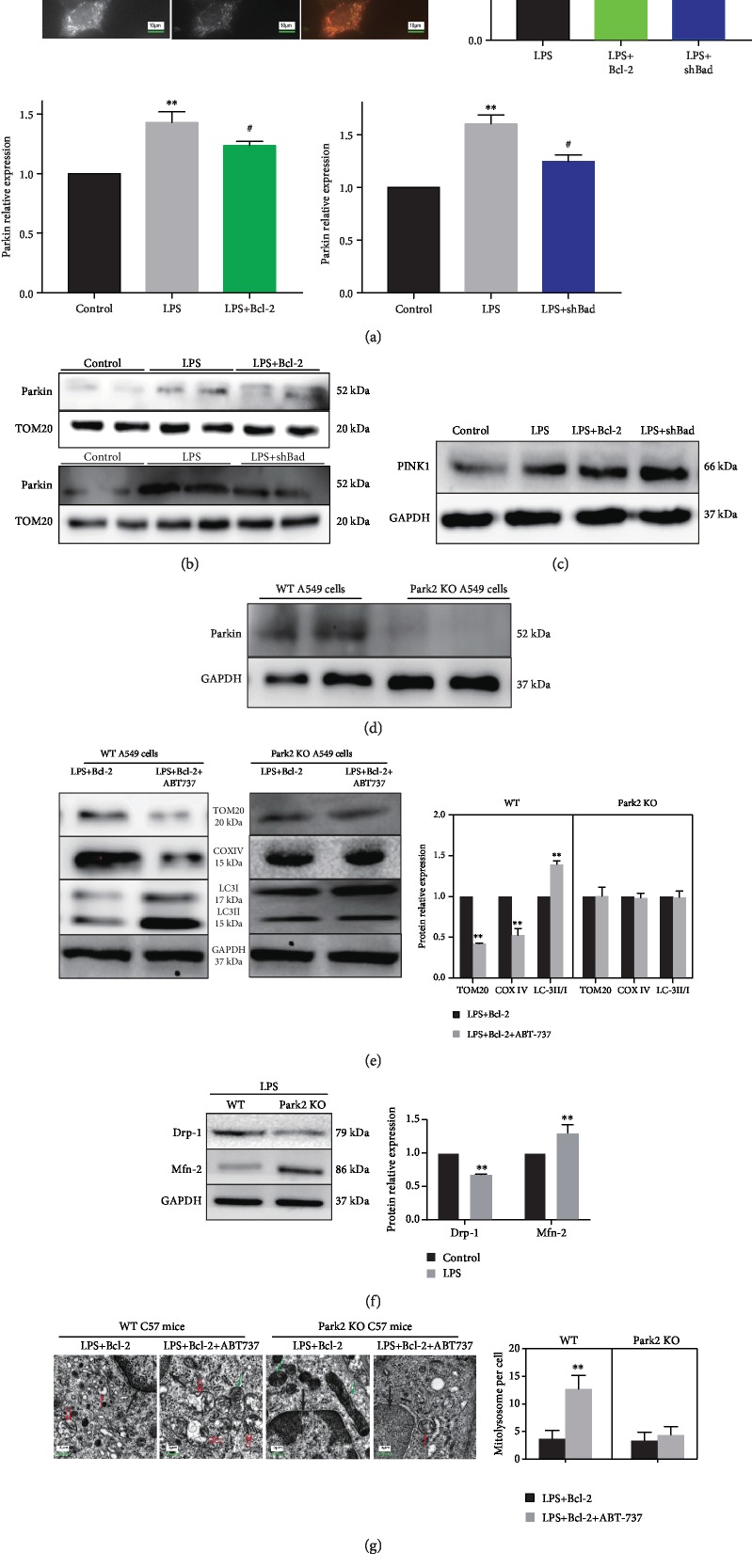
Bcl-2 proteins regulate Parkin recruitment from the cytoplasm to mitochondria. (a) Immunofluorescence showing the reduced colocalization of Tom20 and Parkin in the LPS+Bcl-2 and LPS+shBad groups (*n* = 3). (b) Western blots showing reduced Parkin expression in the mitochondria in the LPS+Bcl-2 and LPS+shBad groups (*n* = 3). (c) Western blots showing that overexpression of Bcl-2 or shBad did not change the expression of PINK1 in the cytoplasm. (d) Western blots showing Parkin expression in the Park2 KO A549 cells or WT A549 cells by using CRISPR/cas9 technology. (e) Western blots showing the expression of Tom20, COX IV, LC3-I, and LC3-II in wild-type A549 cells and Park2 KO cells under LPS+Bcl-2 treatment with or without ABT-737 (a Bcl-2 inhibitor). (f) Western blots showing the expression of Drp1 and Mfn2 in WT A549 cells or Parkin KO A549 cells. (g) Transmission electron microscopy showing mitophagy in lung tissues of wild-type and Park2 KO mice under LPS+Bcl-2 treatment with or without ABT-737 (*n* = 6). Black arrow, nucleus; green arrow, mitochondria; red arrow, mitophagosome; and double red arrow, mitolysosome. (h) Immunoprecipitation assay showing the interaction between Bcl-2 and Parkin as well as between Bcl-2 and Bad in A549 cells exposed to LPS. ^∗∗^*P* < 0.01 vs. the LPS group (a) or vs. the control group (b) or vs. the LPS+Bcl-2 group (e, f); ^#^*P* < 0.05 vs. the LPS group.

To further verify the role of Bcl-2 family proteins in mitophagy, we performed immunofluorescence analysis of Parkin and Tom20 in A549 cells. Both Bcl-2 overexpression and Bad knockdown decreased colocalization of Parkin and Tom20 in LPS-treated cells (Figure 6(a)). In addition, both Bcl-2 overexpression and Bad knockdown reduced mitochondrial expression of Parkin in cells during LPS (Figure 6(b)). However, the PINK1 protein expression was unaffected by Bcl-2 overexpression or Bad knockdown (Figure 6(c)).

CRISPR/cas9-mediated Park2 KO in A549 cells was confirmed by Western blot analysis (Figure 6(d)). To investigate the role of Bcl-2 proteins in Parkin recruitment from the cytoplasm to the mitochondria, we used ABT-737 which acts as a Bad mimic to antagonize Bcl-2 [26]. WT and Parkin KO A549 cells with Bcl-2 overexpression were treated with ABT-737 (5 *μ*M) for 8 h prior to LPS stimulation. In mice, after Bcl-2 transfection, intraperitoneal ABT-737 (20 mg/kg) was given, followed by intratracheal LPS administration. The results showed that ABT-737 reversed the effects of Bcl-2 overexpression on mitophagy *in vitro* (Figure 6(e)) and *in vivo* (Figure 6(g)) in wild-type cells and mice, but not in Park2 KO cells and Park2^−/−^ mice. Parkin interacts with proteins that regulate mitochondrial fission and fusion, such as Drp1 and Mfn1/2, via pathways that depend on the ubiquitin-proteasome system (UPS) [27]. Compared to wild-type A549 cells, Park2 KO induced Mfn2 upregulation and Drp1 downregulation (Figure 6(f)). These findings suggest that Parkin plays a key role in the regulation of mitochondrial fission and fusion. Finally, immunoprecipitation studies revealed the direct interactions between Bcl-2 and Parkin as well as between Bcl-2 and Bad in LPS-treated cells (Figure 6(h)). These results demonstrate that Bcl-2 and Bad directly modulate PINK1/Parkin-mediated mitophagy by regulating the translocation of Parkin from the cytoplasm to the mitochondria in LPS-induced ALI.

## 3. Discussion

In this study, we used LPS to establish ALI models in cells and mice. LPS has been widely used to induce sepsis and systemic inflammation during ALI [27]. Mitochondria play a crucial role in cell metabolism, growth, proliferation, differentiation, and cellular responses to stress. The functional status of mitochondria determines cell survival and death [28]. Mitophagy is an autophagic process to maintain mitochondrial hemostasis [29–31]. In LPS-induced ALI, damaged mitochondria were removed by mitophagy, reducing ROS production and attenuating ALI [32, 33]. However, excessive mitophagy may also be detrimental [28], and the detailed mechanisms are still unclear. In our previous study, we found that inhibition of mitophagy with mitochondrial division inhibitor 1 significantly reduced ALI in rats [34]. In this study, we showed that excessive mitophagy resulted in impaired ATP production, insufficient cell energy supply, and increased ROS levels, which have been reported to damage mitochondrial membrane permeability and the electron transport chain (ETC) [29–31]. Moreover, in Atg5 KO cells, we found that cell apoptosis was significantly alleviated by inhibition of mitophagy. The mitochondrial membrane potential dissipation and ETC inhibition lead to decreased mitochondrial ATP production and increased mito-ROS release, promoting mitophagy. Therefore, mitophagy promoted LPS-induce ALI in our study, but not maintaining the normal mitochondrial function or cell homeostasis.

In addition to a scattered distribution of small and bean-shaped mitochondria in the cytoplasm, long tubular mitochondria interconnect to form mitochondrial networks in various cell types [35]. The intact mitochondrial reticular structure is crucial for mitochondrial and cellular function [21]. Highly dynamic remodeling of mitochondrial networks induced by stress has been observed [22]. Under mild stress conditions, damaged mitochondria are separated from the network and eliminated by mitophagy. Under conditions of severe injury, such as sepsis, mitochondrial reticular structure disorder leads to mitochondrial membrane potential dissipation and cell death [36]. Mfn2 and Drp1 which participate in the process of mitochondrial division and fusion play an important regulatory role in the dynamic balance of the mitochondrial network structure [21, 37]. In this study, we observed that the mitochondrial network was severely disrupted during LPS-induced ALI, with downregulation of Mfn2 and upregulation of Drp1. Our results for the first time showed that LPS-induced mitophagy caused disrupted mitochondrial network structure, a sharp decrease in ATP supply, and increased mito-ROS production, while these changes are alleviated by inhibition of excessive mitophagy.

Bcl-2 family proteins play a key role in mitochondrial homeostasis [35]. Bcl-2, containing four highly conserved domains BH 1–4, functions as an antiapoptotic protein by regulating mitochondrial membrane permeability and cytochrome C release [17, 38]. Voehringer and Meyn reported that Bcl-2 overexpression reduced intracellular ROS levels and oxidative stress-related cell damage [17, 38]. In addition, Bcl-2 family proteins have been shown to regulate mitochondrial morphogenesis, autophagy, and ROS elimination [39–41]. Other studies revealed that Bcl-2 regulates calcium homeostasis and endoplasmic reticulum stress [39–41]. However, the role of Bcl-2 and Bad (two Bcl-2 family proteins) in the regulation of mitophagy in LPS-induced ALI is unclear. To further investigate the mechanism, we established the LPS-induced *in vitro* and *in vivo* ALI model to show the involvement of Bcl-2 and Bad in this process. Furthermore, we demonstrated that both Bcl-2 overexpression and Bad knockdown attenuated LPS-induced cell injury and mice ALI by inhibiting mitophagy. Bcl-2 family proteins are involved in the fission and fusion of mitochondria [21]. In this study, we have shown that Bcl-2 upregulation and Bad downregulation participate in the maintenance of the integrity of the mitochondrial reticular structure during LPS-induced ALI. In addition, we also found that the other members of the Bcl-2 family including Bcl-xl, Bcl-w, and Bim took part in this process.

The mitochondrial serine/threonine protein kinase PINK1 is stabilized on impaired mitochondria during mitochondrial depolarization, recruiting Parkin from the cytoplasm to the mitochondrial membrane to initiate mitophagy [21]. As expected, we found increased PINK1 protein expression in LPS-treated A549 cells. Furthermore, immunofluorescence analysis revealed colocalization of Parkin and Tom20 after LPS treatment and analysis of isolated mitochondria confirmed the location of the upregulated Parkin. These results suggest that Parkin is recruited from the cytoplasm to mitochondria in LPS-induced ALI and mitophagy is activated by LPS via the PINK1/Parkin pathway. To further explore the roles of Bcl-2 and Bad, we generated Park2 KO A549 cells using the CRISPR-Cas9 gene editing technique. We found reduced mitophagy of KO cells than that of wild-type cells following LPS treatment regardless of the presence or absence of Bcl-2 upregulation. Moreover, the antimitophagy function of Bcl-2 was antagonized by treatment with the Bad mimic ABT-737. Conversely, mitophagy was reduced in Park2 KO A549 cells in the presence or absence of ABT-737-mediated Bcl-2 antagonism. Letsiou et al. reported that Parkin KO mice were protected against LPS-mediated ALI compared to wild-type mice and that Parkin modulated mitochondrial autophagy by regulating inflammation-related protein expression and signaling pathways [42]. In this study, we have revealed for the first time that PINK/Parkin-mediated mitophagy is involved in LPS-induced ALI. Similarly, we observed that the number of mitochondrial autophagosomes in Parkin KO mice was significantly lower than that in WT mice. These findings provide evidence that Bcl-2 regulates mitophagy via the PINK1/Parkin signaling pathway in LPS-induced ALI. Interestingly, neither Bcl-2 nor Bad influenced PINK1 stability. On the other hand, Parkin catalyzes the ubiquitination of Mfn2, targeting it for degradation [42]. We found that Mfn2 expression was upregulated in Parkin KO cells. Combining this finding with our previous results indicated that upregulated Bcl-2 caused Mfn2 recovery, possibly by Bcl-2-mediated regulation of Parkin. Parkin ubiquitinates Drp1 for proteasome-dependent degradation [43]; however, it has also been reported that Drp1 expression is downregulated following Parkin KO [44]. Therefore, it can be speculated that Parkin mediates bidirectional regulation of Drp1. In this study, we found that Drp1 expression was downregulated by Parkin KO. In this case, Parkin might induce Drp1 degradation via ubiquitination [45] or promote the recruitment of Bax to the mitochondria [46] to promote mitochondrial fission [21]. We also propose another hypothesis in which Parkin knockdown protects the mitochondrial network structure and membrane potential by inhibiting excessive mitophagy, thereby inhibiting Drp1 expression; however, the specific mechanism remains to be elucidated. Coimmunoprecipitation studies showed that Bad binds directly to Bcl-2 and the latter binds directly to Parkin. Taken together, our findings demonstrate the involvement of Bcl-2 family proteins in regulating mitophagy during LPS-induced ALI through targeting the PINK1/Parkin signaling pathway.

We found that both Bcl-2 overexpression and Bad knockdown reduced mitophagy via the PINK1/Parkin signaling pathway in LPS-induced ALI. However, the potential regulatory effects of other Bcl-2 family proteins on mitophagy induced by LPS and whether the PINK1/Parkin pathway is also involved need to be investigated. Besides, it should be noted that the Bad mimic ABT-737 may antagonize other Bcl-2 protein family proteins and related signaling pathways [47]. In addition, our results obtained in the A549 cell line and mouse models may not be fully extrapolated to humans.

**Figure 7:**
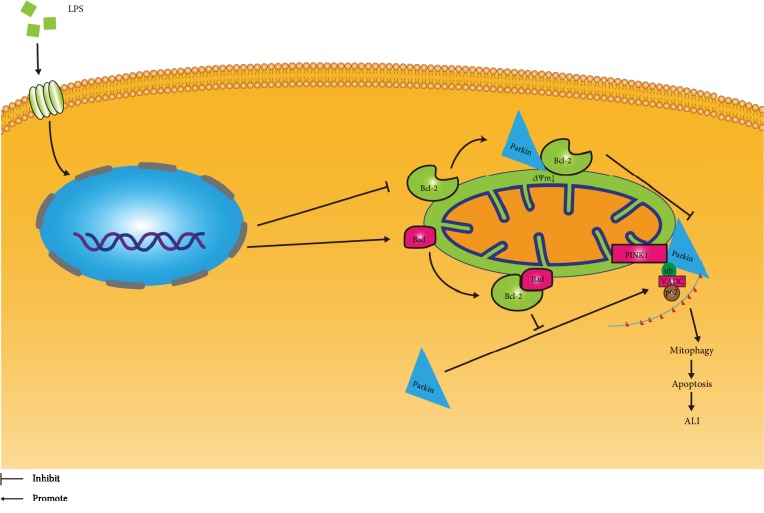
Schematic diagram representing mitophagy and altered expression of mitophagy regulators in LPS-induced ALI. Under LPS stimulation, Bcl-2 expression is downregulated and Bad expression is upregulated. With mitochondrial membrane potential dissipation, cell apoptosis and mitophagy were initiated through the activation of the PINK1/Parkin pathway, leading to ALI. Bcl-2 functions to inhibit the recruitment of Parkin from the cytoplasm to the mitochondria by direct interaction with Parkin, while Bad exerts direct inhibition of the antimitophagic protein Bcl-2.

In conclusion, this is the first study to demonstrate that the Bcl-2 proteins regulate mitophagy in LPS-induced cell injury and mice ALI via direct interactions with the PINK1/Parkin signaling pathway (Figure 7). Based on these results, the Bcl-2 protein family is implicated as a therapeutic target in sepsis-induce ALI.

## 4. Materials and Methods

### 4.1. Cell Culture

A549 cells were purchased from the KeyGEN BioTECH (Jiangsu, China). Cells were cultured in Dulbecco's modified Eagle medium/F12 (DMEM/F12) (Gibco, Grand Island, NY, USA) supplemented with 10% fetal bovine serum (FBS) (Gibco), 100 U/ml penicillin, and 100 *μ*g/ml streptomycin at 37°C under 5% CO_2_.

### 4.2. Animals

Animal experiment protocols were approved by the Animal Welfare and Ethics Management Committee of Southern Medical University (China). All experiments were conducted in accordance with the Guide for the Care and Use of Laboratory Animals. C57BL/6 mice (male, aged 8–10 weeks, 18–20 g) were purchased from the Center of Experimental Animals of Guangdong Province. Two pairs of Park2^−/−^ C57BL/6 mice (15 days) were purchased from Saiye Biotechnology Co., Ltd. (Jiangsu, China). The offspring mice at 8–10 weeks old were selected for experiments. All mice received standard care in a controlled environment (12 h light/dark cycle, 22°C), with free access to food and water.

### 4.3. Experimental Models

For the *in vitro* model, LPS (L2880, Sigma, St. Louis, MO, USA) was diluted with phosphate-buffered saline (PBS). The A549 cells were washed with PBS, and LPS was added to the cell culture medium. For the *in vivo* model, LPS was diluted with normal saline. The main trachea was exposed through a neck incision. Mice in the LPS group received intratracheal LPS delivery using a 1 ml syringe and were kept in the upright position for 10 min. Mice in the control group received the same volume of normal saline.

### 4.4. Cell Viability and Cytotoxicity Assays

Cell viability was assessed by using the Cell Counting Kit-8 (CCK8) assay (Dojindo, Japan), and cytotoxicity was assessed by using the lactate dehydrogenase (LDH) assay kit (Beyotime, China) according to the manufacturer's instructions [32, 48]. The absorbance values at 490 nm were measured using a microplate reader (Biotek, Winooski, VT, USA).

### 4.5. ROS and ATP Measurement

Reactive oxygen species (ROS) were measured by flow cytometric detection of DCFH2-DA (Jiancheng Biotechnology, China), following the manufacturer's instructions. Intracellular adenosine 5′-triphosphate (ATP) concentration was analyzed using the EnzyLight™ ATP Assay Kit (BioAssay Systems, Hayward, CA, USA).

### 4.6. Mitochondrial Membrane Potential Detection

Mitochondrial membrane potential was detected using the JC-1 kit (Jiancheng Biotechnology, China) according to the manufacturer's instructions. In healthy cells, JC-1 aggregates are detected by red fluorescence (485/535 nm). In apoptotic cells, JC-1 monomers are detected by green fluorescence (550/600 nm). The ratio of aggregates (red) to monomers (green) was calculated using the microplate reader (BioTek).

### 4.7. Wet/Dry Ratio

After the experiment, the lungs were excised and blotted, and the wet weight was measured. The lungs were then desiccated at 80°C for 48 h to obtain the dry weight. To evaluate lung tissue edema, the wet/dry weight ratio was calculated.

### 4.8. Protein in Bronchoalveolar Lavage Fluid (BALF)

The bronchoalveolar lavage was repeated three times with 1 ml PBS through the endotracheal tube. The BALF was collected and centrifuged at 2500 rpm for 10 min, and the protein concentration in the supernatant was measured by using the BCA method [49].

### 4.9. Hematoxylin and Eosin (HE) Staining and Lung Injury Score

The lungs were fixed with 10% buffered formalin, embedded with paraffin, and cut into sections. Images were captured under a microscope (Olympus Tokyo, Japan). Lung injury scores were calculated by using the pathological ALI scoring system from the American Thoracic Association [18]. This system comprises four categories (alveolar congestion, hemorrhage, infiltration or aggregation of neutrophils in the alveolar cavity or vascular wall, and alveolar wall thickening and/or transparent membrane formation), with a score of 0 to 4 in each domain (0, no lesions or very mild lesions; 1, mild lesions; 2, moderate lesions; 3, severe lesions; and 4, extremely severe lesions) and a total score of 16. Lung injury was evaluated by independent pathologists who were blinded to the group allocation.

### 4.10. Mitochondrial Extraction

Mitochondria were extracted by using a mitochondrial extraction kit (KeyGEN BioTECH, China) according to the manufacturer's recommendations. In brief, A549 cells were digested and washed with PBS three times. After centrifugation at 800 × *g* for 5 min, the cells were collected. Reagents were added to 10^7^ cells to obtain the mitochondrial suspension. Finally, the mitochondrial suspension and the cytoplasmic fraction were stored at -80°C, respectively.

### 4.11. Mitophagy Evaluation

Mitophagy was evaluated by cotransfection with adenovirus GFP-LC3 vector (AD-GFP-LC3) and adenovirus mitochondrial specific localization fluorescence probe (hbad-mito-dsred) for 24 h in cells cultured in confocal dishes [5]. The colocalization was observed under a fluorescence microscope (Olympus Tokyo, Japan). The expression of mitochondrial-related proteins (Tom20, COX IV, Mfn2, and Drp1) and autophagy-related proteins (LC-3) was also detected by Western blot analysis [50–52].

### 4.12. Western Blot Analysis

Proteins were resolved by 10% sodium dodecyl sulfate-polyacrylamide gel electrophoresis (SDS-PAGE), transferred to polyvinylidene fluoride (PVDF) membranes, and blocked in 5% skimmed milk for 1 h. The membranes were then incubated overnight at 4°C with the following primary antibodies (anti-rabbit or anti-mouse): cleaved caspase3 (1 : 1,000; Affinity, China), Tom20 (1 : 1,000; BD, USA), COXIV (1 : 1,000; Affinity, China), LC3B (1 : 500; Affinity, China), P62 (1 : 1,000; Affinity, China), Bcl-2 (1 : 1,000; Affinity, China), Bad (1 : 1,000; Affinity, China), Parkin (1 : 1,000; Proteintech, USA), PINK1 (1 : 1,000; Proteintech, USA), Drp1 (1 : 1,000; Affinity, China), Mfn2 (1 : 1,000; Affinity, China), Atg-5 (1 : 800; Affinity, China), *β*-actin (1 : 5,000; Affinity, China), GAPDH (1 : 5,000; Affinity, China), Bcl-xl (1 : 1,000; Affinity, China), Bcl-w (1 : 1,000; Affinity, China), Bim (1 : 1,000; Affinity, China), procaspase3 (1 : 1000; Abcam, USA), and Tubulin (1 : 5,000; Affinity, China). Membranes were then incubated for 2 h with horseradish peroxidase-conjugated anti-rabbit or anti-mouse IgG secondary detection antibodies (1 : 5,000). Immunoreactivity was detected by using an enhanced chemiluminescence detection system (Beyotime, Haimen, China) and visualized on an imaging system (Kodak, Shanghai, China).

### 4.13. Immunohistochemistry

Bcl-2 and Bad expression in lung tissues was visualized by using commercial immunohistochemistry kits (Beyotime, Haimen, China) according to the manufacturer's instructions. In brief, tissue sections were heated at 65–75°C for 1.5 h. Sections were then dewaxed by immersion in xylene for 10 min. Sections were then rehydrated in a graded ethanol series (100%, 100%, 95%, and 80%, diluted in purified water), each for 5 min. After antigen retrieval, tissues were permeabilized with 0.5% Triton X-100/PBS for 20 min at room temperature. To eliminate endogenous peroxidase activity, sections were incubated in freshly prepared 3% hydrogen peroxide blocking solution for 10 min at room temperature. After blocking, the sections were incubated overnight at 4°C with primary detection antibodies (anti-Bcl-2, 1 : 50, Affinity; anti-Bad, 1 : 50, Affinity). The sections were then incubated with appropriate secondary antibodies (1 : 100) for 1 h at room temperature. The staining was visualized under the microscope. Sections were then washed and restained with hematoxylin for 3 min and differentiated with hydrochloric acid/alcohol. Sections were then rinsed, dehydrated, and visualized under the microscope.

### 4.14. Immunofluorescence

After washing with preheated PBS, cells were treated with 4% formaldehyde for 30 min at room temperature. Cells were permeabilized with 0.2% Triton X-100 (Beyotime, Haimen, China) for 2–5 min, washed three times, and blocked with 5% BSA for 30 min at room temperature. Cells were then incubated overnight at 4°C with the following primary antibodies: anti-Parkin, anti-Tom20, and anti-LC-3 (diluted with 1% BSA). Then, cells were incubated with the secondary antibody (1 : 50), washed with PBS, and sealed with 95% glycerin. Immunofluorescence was observed under a fluorescence microscope (Olympus Tokyo, Japan). Finally, Pearson's correlation coefficient was calculated to evaluate the colocalization of target proteins.

### 4.15. Transmission Electron Microscopy

The lung tissues were fixed with 2.5% glutaraldehyde for 4 h and washed with PBS. After treatment with 1% osmium tetroxide for 2 h, the tissues were dehydrated using gradient alcohol series (50%, 70%, 80%, and 90%; 10–15 min each), followed by 100% alcohol (10–15 min, twice). Tissues were then immersed in epoxy propane soaking/embedding solution (3 : 1) for 30 min, epoxy propane/embedding solution (1 : 1) for 3 h, and pure embedding solution overnight at room temperature. After being embedded in the capsule or embedding plate with the embedding agent, samples were incubated at 37°C for 24 h followed by 60°C for 48 h. Ultrathin sections (100 nm) were then prepared using microtome Leica UC7 (Leica, Wetzlar, Germany). The sections were stained with uranium dioxide acetate for 20 min, followed by lead citrate staining for 10 min. Finally, the autophagosomes were photographed under a transmission electron microscope (Nippon Electronics Co., Japan).

### 4.16. Bcl-2 Overexpression and Bad Knockdown

The adenovirus vector for Bcl-2 overexpression (AAV-CMV-Bcl-2) was purchased from Guanzi Biotechnology Co., Ltd. (Hangzhou, China). The short hairpin RNA of Bad (ShBad) lentivirus vector was purchased from Suzhou GenePharma Biotechnology Co., Ltd. (Suzhou, China). The vector was diluted with PBS. A549 cells were transfected with adenovirus (100 MOI) for 36 h, and the expression of Bcl-2 or Bad was assessed. Mice were injected with adenovirus (10^11^/ml) through the tail vein, and Bcl-2 or Bad protein expression was determined at 14 days.

### 4.17. Gene Knockout Cells

The CRISRP/Cas9 system was used to construct the Park2 (Parkin) KO or Atg5 KO A549 cell line. KO cells were obtained through transfection, screening, and cell monoclonal culture. Park2 gRNA sequences were as follows: GTGTCAGAATCGACCTCCAC (Park2-gRNA-1#) and CAGTTGCGTGTGATTTTCGC (Park2-gRNA-2#). Sequencing primers were as follows: CACTGAAGGGCTGCGAGGGG (forward) and TTGTGCCTTGCTGCTCCTGT (reverse). The Atg5 sgRNA was located behind an U6 promoter and contained the following target sequence: 5′-GTGCTTCGAGATGTGTGGTTTGG-3′ or 5′-AAGATGTGCTTCGAGATGTGTGG3′.

### 4.18. Reverse Transcription Quantitative Polymerase Chain Reaction (RT-qPCR)

Total RNA was extracted with the Trizol reagent (GeneCopoeia, MD, USA). Complementary DNA (cDNA) was synthesized. RT-qPCR assays were performed using the PCR (Thermo Fisher Scientific, Waltham, MA, USA) and LightCycler96 system (Roche Basel, Switzerland) with the following specific primers: Bcl-2 (forward 5′-GGAGAGTGCTGAAGATTGA-3′ and reverse 5′-GTCTACTTCCTCTGTGATGT-3′), Bad (forward 5′-GGAGGATGAGTGACGAGTT-3′ and reverse 5′-TCCTCTCCCCAAGTTCCGAT-3′), and PINK1 (forward 5′-ACAGGCTCACAGAGAAGT-3′ and reverse 5′-AGACCATCACGACACAGA-3′).

### 4.19. Statistical Analysis

Data were represented as the mean ± standard error of the mean (SEM) and analyzed using the SPSS 20.0 (California, USA). Student's *t*-test or analysis of variance (ANOVA) was used as appropriate. Two-tailed *P* values of <0.05 were considered to indicate statistical significance.

## Data Availability

The data used to support the findings of this study are available from the corresponding author upon request.

## Abbreviations

LPS: Lipopolysaccharide
ALI: Acute lung injury
PARL: Presenilin-associated rhomboid-like protease
ROS: Reactive oxygen species
PBS: Phosphate-buffered saline
BALF: Bronchoalveolar lavage fluid
ATS: American Thoracic Society
shBad: Short hairpin RNA of Bad
ETC: Electron transport chains
UPS: Ubiquitin-proteasome system
Mfn2: Mitofusin-2
Drp1: Dynamin-related protein 1.

## Supplementary Materials

Supplementary 1 Figure S1. Atg5 KO inhibited LPS-induced cell apoptosis.

Supplementary 2 Figure S2. Protein expression of Bcl-xl, Bcl-2, and Bim in LPS-treated cells.
