# Supplementary figures and images for: Bcl-2 Proteins Regulate Mitophagy in Lipopolysaccharide-Induced Acute Lung Injury via PINK1/Parkin Signaling Pathway
Source: Oxid Med Cell Longev. 2020 Feb 20;2020:6579696. doi: 10.1155/2020/6579696 (PMC7054785; doi:10.1155/2020/6579696)

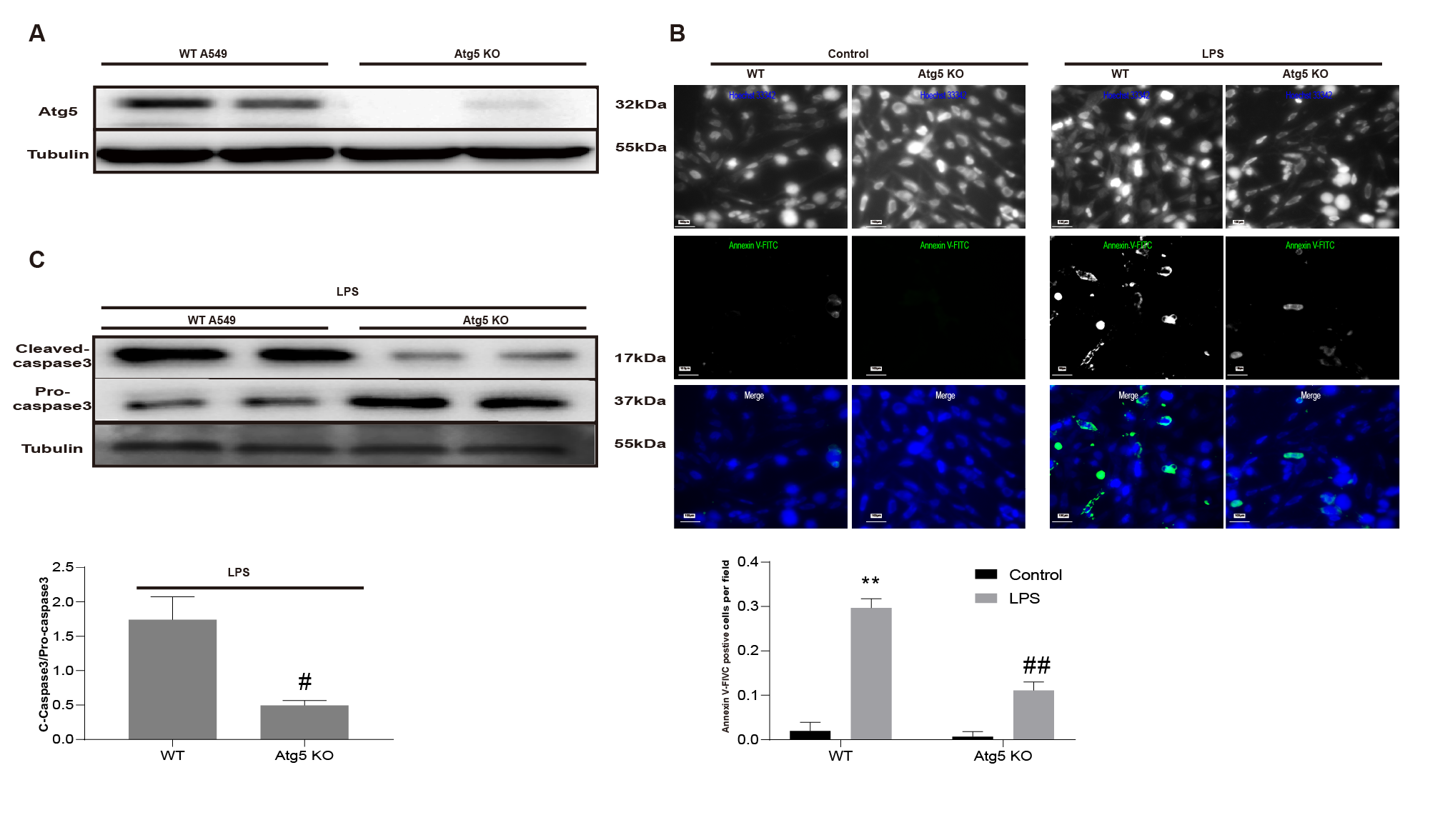

Supplement: Supplementary 1 — Figure S1. Atg5 KO inhibited LPS-induced cell apoptosis. [file 6579696.f1.tif]

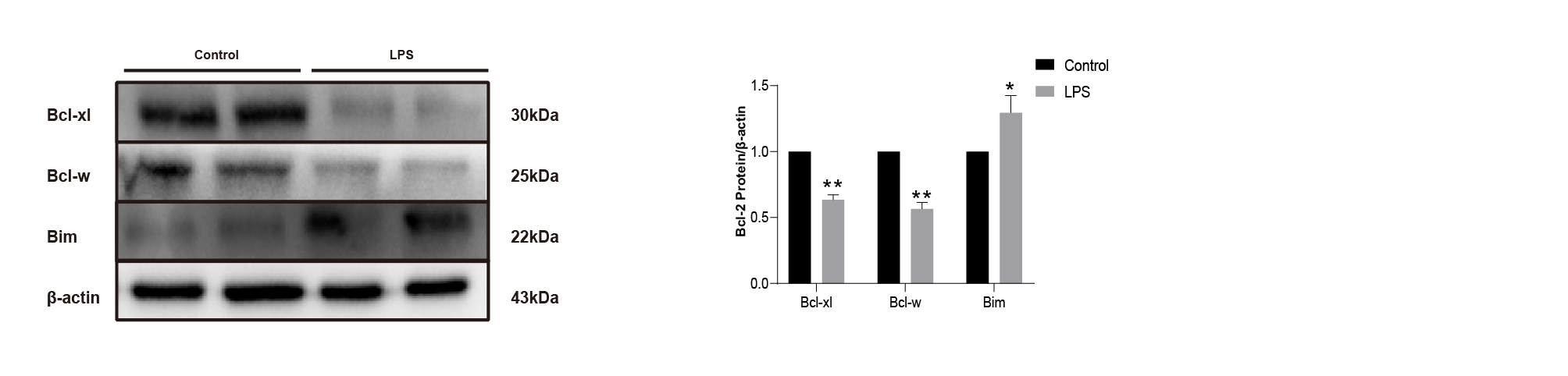

Supplement: Supplementary 2 — Figure S2. Protein expression of Bcl-xl, Bcl-2, and Bim in LPS-treated cells. [file 6579696.f2.tif]
